# PDGFRA, HSD17B4 and HMGB2 are potential therapeutic targets in polycystic ovarian syndrome and breast cancer

**DOI:** 10.18632/oncotarget.17846

**Published:** 2017-05-13

**Authors:** Huiyu Xu, Yong Han, Jiaying Lou, Hongxian Zhang, Yue Zhao, Balázs Győrffy, Rong Li

**Affiliations:** ^1^ Department of Obstetrics and Gynecology, Reproductive Medical Center, Peking University Third Hospital, Beijing, P.R. China; ^2^ Department of Pathology, Zhejiang Provincial People’s Hospital, Hangzhou, Zhejiang Province, P.R. China; ^3^ Department of Clinical Laboratory, Renmin Hospital of Xiaoshan District, Hangzhou, Zhejiang Province, P.R. China; ^4^ Department of Urology, Peking University Third Hospital, Beijing, P.R. China; ^5^ Momentum Cancer Biomarker Research Group, Research Centre for Natural Sciences, Hungarian Academy of Sciences, Budapest, Hungary; ^6^ Second Department of Pediatrics, Semmelweis University, Budapest, Hungary

**Keywords:** PCOS, obese, insulin-resistant, muscle, breast cancer

## Abstract

To explore the key genes associated with both PCOS and breast cancer, we overlapped the synchronously differently expressed genes in two obese insulin-resistant GEO datasets in muscle tissue and genes exert essential roles in breast cancer prognosis together base on the following reasons: (1) Androgens excess is believed to contribute to the onset of both PCOS and breast cancer. (2) PCOS is usually complicated with metabolic symptoms, such as obesity and insulin-resistance. (3) Muscle is the main place where energy metabolism and material metabolism take place. Consequently, 53 genes were found, functionally enriched in pathways such as pyruvate metabolism, muscle system process and development of primary male sexual characteristics etc. We further lay our eyes on genes correlated with male sexual characteristics, which may be involved in the onset of both PCOS and breast cancer. Three genes were indicated to be associated with this process, including hydroxysteroid (17-beta) dehydrogenase 4/HSD17B4, platelet-derived growth factor receptor, alpha polypeptide/PDGFRA and high-mobility group box 2/HMGB2. Gene-drug interaction network about the three genes were then constructed. Drugs or chemicals that contribute to correcting the disorder of lipid metabolism were detected to restore the abnormal expression of the three genes in PCOS, such as simvastatin, bezafibrate, fenofibrate et al, which provide further choices for managing patients with PCOS.

## INTRODUCTION

Polycystic ovary syndrome, which is short for PCOS, is a highly complex endocrine and metabolic disorder, characterized by clinical and/or biological signs of androgen excess with prolonged menstrual cycle, oligo ovulation or anovulation, hirsutism, polycystic ovarian, and high level of circulating androgens, and is frequently associated with insulin resistance and obesity. Hyperandrogenism and/or hyperandrogenemia are suggested to contribute to the pathogenesis of PCOS [1, 2]. Nevertheless, the insight mechanism is still largely unknown.

The pathogenesis of breast cancer, is initiated by excessive androgen and estrogen. Large amount of data has proved that high serum level of androgens and estrogens were positively correlated with increased risk of breast cancer incidences and breast cancer recurrences [3–13]. Since androgen excess could contribute to the pathogenesis of both PCOS and breast cancer, there might be an overlap between dysregulated genes in PCOS and genes associated with breast cancer.

In this study, we first choose the commonly different expressed genes in two GEO datasets of obese insulin-resistant PCOS women in muscle tissue and then overlap them with genes that has prognostic value in breast cancer. Then, function enrichment analysis was performed on these overlapped genes. Gene correlated with androgen metabolism and male sexual characteristics were selected for further analysis. Finally, gene-drug interaction network between selected genes and drugs or chemicals that could result in their decreased or increased expression was constructed.

## RESULTS

### Potent key genes in pathogenesis of PCOS

**Figure 1:**
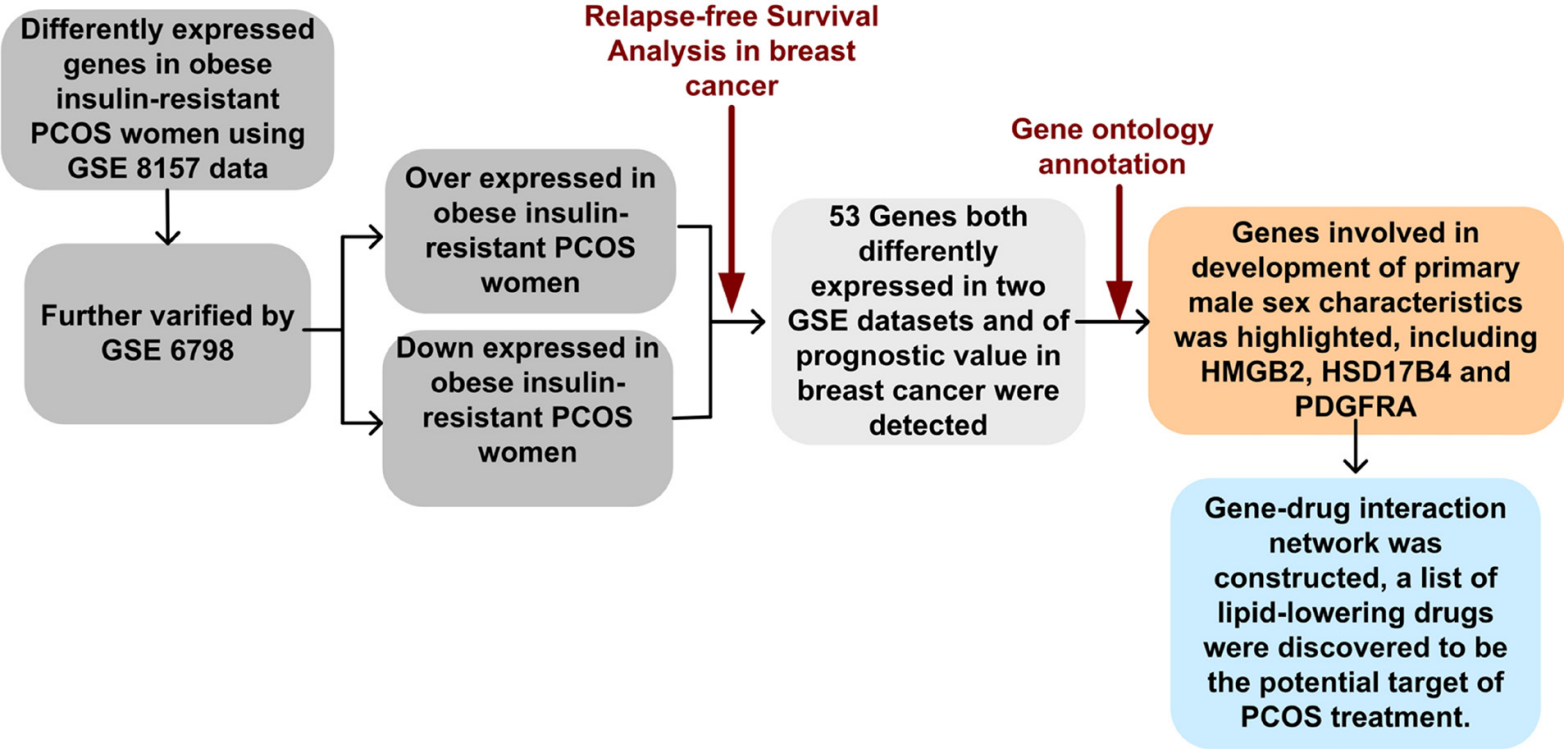
Flow chart of this study First, two PCOS datasets were selected from Gene Expression Omnibus for screening common genes associated with PCOS. Then, the prognostic value of these genes were evaluated using our merged breast cancer dataset which containing 3554 samples. Next, function enrichment analysis of selected genes were performed. In the end, gene-drug interaction network was constructed and drugs that could potentially influencing the three genes were indicated.

It is well known that PCOS and breast cancer are both correlated with disorder of sex hormone metabolism, and they are both metabolic disease. Since high level of androgens and estrogens have been proved to positively correlated with increased risk of breast cancer, we sought to overlap dysregulated genes in PCOS with prognostic genes in breast cancer to find common therapeutic targets of PCOS and breast cancer. The schematic structure of the article was shown in Figure 1.

Two publicly available datasets of gene expression profile in skeletal muscle of insulin-resistant obese PCOS women and their age- and body mass index matched corresponding healthy controls were downloaded and analyzed to explore the commonly different-expressed genes in both GSE datasets. Both datasets were completed at the platform of Affymetrix Human Genome U133 Plus 2.0 Array.

Disorder of sex hormone metabolism contribute to onset of both PCOS and breast cancer. Besides, PCOS is suggested to be the risk factor for breast cancer [14]. Thus, we further performed the Kaplan-Meier analysis in 3554 breast cancer patients in different stages using the differently expressed genes in both GEO datasets. Those genes differently expressed in both datasets and have influence on breast cancer prognosis were hypothesized to be the key genes in pathogenesis of PCOS (Supplementary Table 1). Of the 60 probe sets, 53 genes were discovered.

### Gene ontology analysis of potential key genes in PCOS

Gene ontology analysis of the 53 potent key genes in pathogenesis of PCOS was carried out. To our interest, several well-known processes involved in PCOS were discovered in our top-ranked gene ontology processes, such as pyruvate metabolic process, and development of primary male sexual characteristics etc. (Table 1).

**Table 1:** Gene ontology analysis of 53 key genes

GO Terms	Genes	*p*-Value
pyruvate metabolic process	SLC16A3, LDHB, SLC16A7	7.10E-03
muscle system process	CALD1, RYR3, MYH4, MSTN	1.40E-02
development of primary male sexual characteristics	HMGB2, PDGFRA, HSD17B4	1.70E-02
male sex differentiation	HMGB2, PDGFRA, HSD17B4	2.10E-02
response to activity	MYH4, MSTN	3.60E-02
organic acid catabolic process	ALDH6A1, AASS, HSD17B4	4.40E-02
protein import into nucleus, docking	IPO4, TNPO2	5.00E-02
nucleobase metabolic process	ALDH6A1, AMPD3	6.70E-02
organic acid transport	SLC16A3, SLC16A7, SLC38A1	7.40E-02
sex differentiation	HMGB2, PDGFRA, HSD17B4	7.70E-02

### Genes suggested in development of primary male sexual characteristics

**Figure 2:**
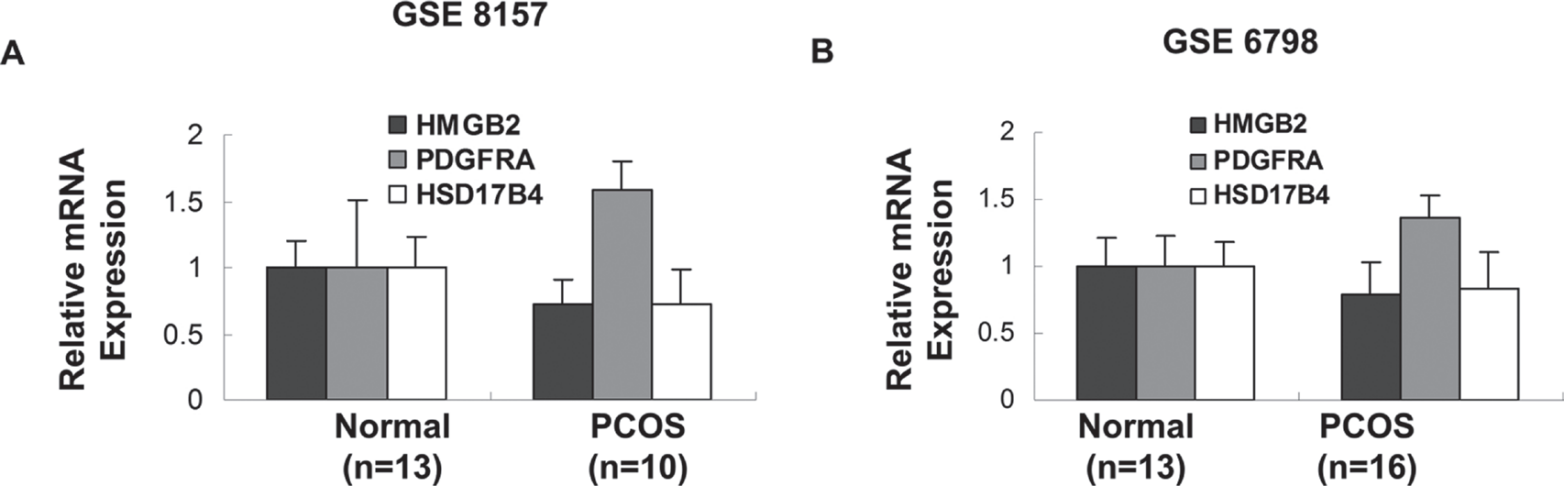
Genes involved in development of primary male sex characteristics The expression of HMGB2, PDGFRA and HSD17B4 in insulin-resistant obese PCOS women and their age- and body mass index- matched controls in two different GEO datasets were demonstrated. (*p* values were calculated by unpaired two-tailed *t*-test, error bars represent mean ± SEM).

**Figure 3:**
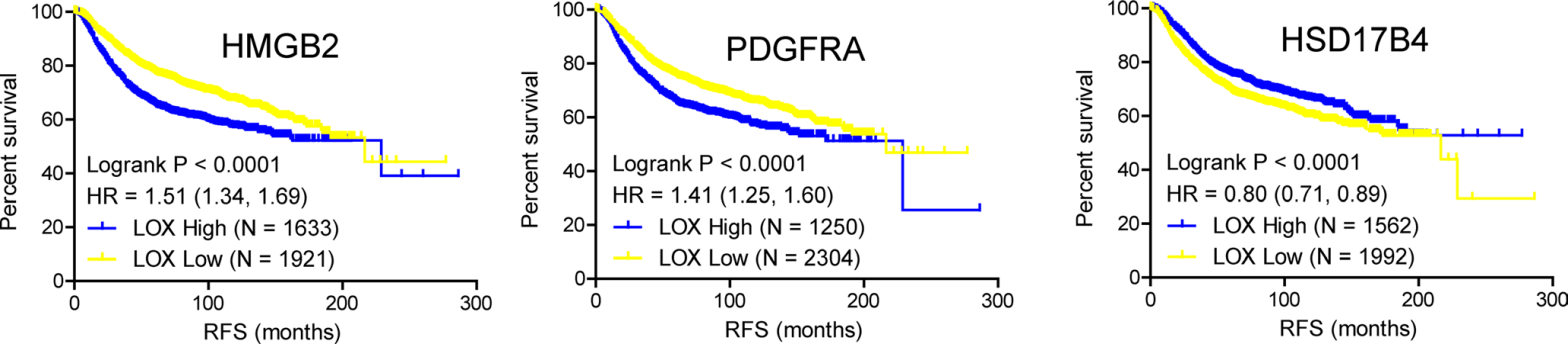
Kaplan-Meier plot of the relapse free survival of 3554 breast cancer patients based on HMGB2, PDGFRA and HSD17B4, respectively (*p* value was calculated by Logrank test, significance was defined as *p* < 0.05).

PCOS, characterized by elevated male sex hormone levels, is one of most common endocrine disorder in women of reproductive age. Of the 53 potent key genes in pathogenesis of PCOS, HMGB2, PDGFRA and HSD17B4 were demonstrated in the development of male sexual characteristics or male sex differentiation. HMGB2 and HSD17B4 were down-regulated in obese PCOS women, while PDGFRA was up-regulated in obese PCOS women (Figure 2A, 2B). In terms of prognosis of breast cancer, high expression of HMGB2 and PDGFRA in breast cancer indicated poor prognosis, while high expression of HSD17B in breast cancer suggested the opposite effect (Figure 3). The above results indicated that reduced expression of HSD17B4 and increased expression of PDGFRA in PCOS may be a risk factor of PCOS developing into breast cancer. While the decreased expression of HMGB2 may be the compensatory response the body correspond to PCOS.

### Construction of network between chemicals and genes involved in development of male sexual characteristics

**Figure 4:**
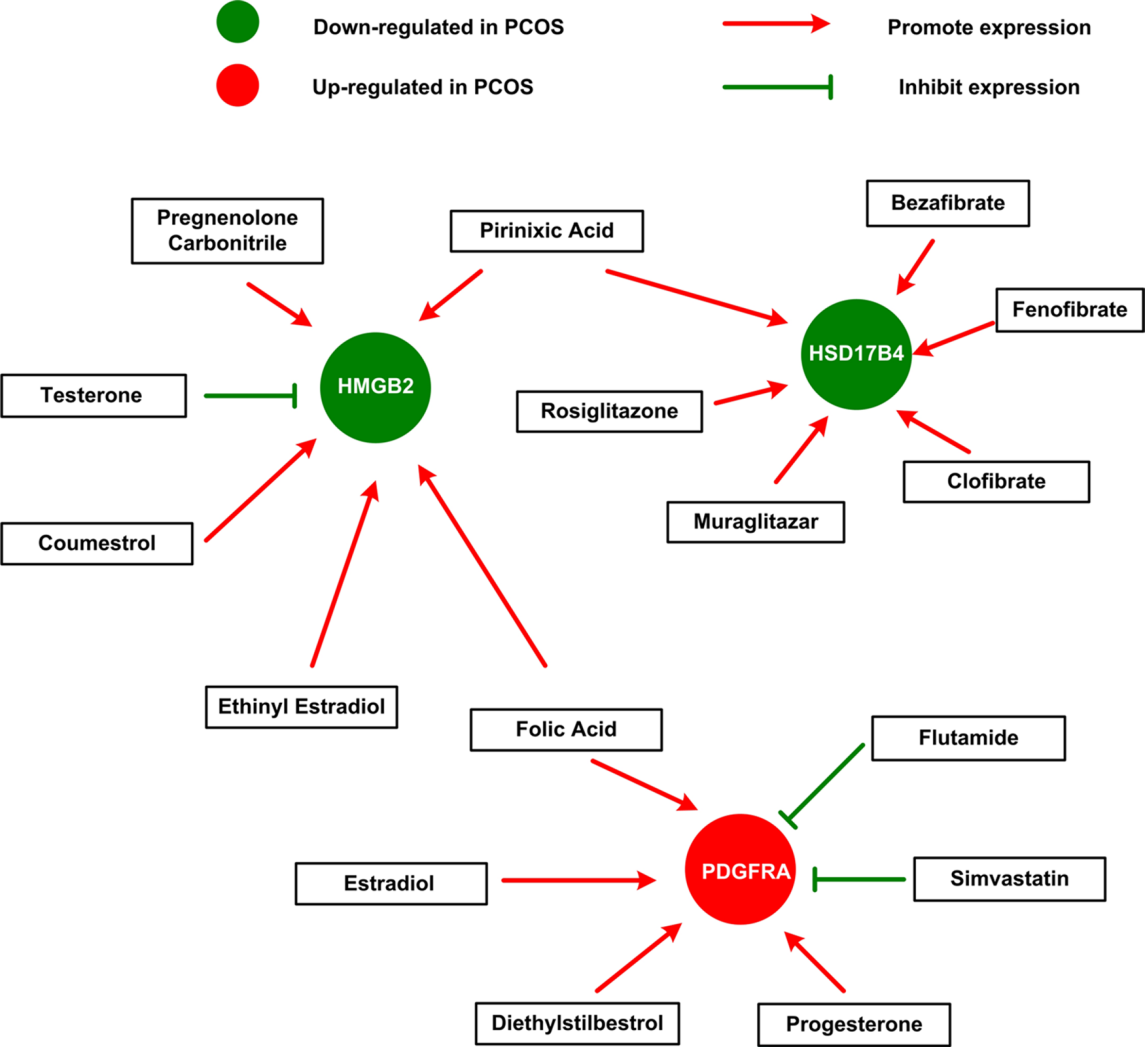
The gene-drug interaction network The network shows us how available drugs or chemicals could decrease or increase the expression of HMGB2, PDGFRA and HSD17B4. For example, simvastatin could decrease the expression of PDGFRA, while estradiol could increase the expression of PDGFRA.

Next, we sought to explore how HMGB2, PDGFRA and HSD17B4 and available chemicals or drugs could influence each other. Gene-drug interaction network was constructed based on data from The Comparative Toxicogenomics Database (CTD) (Figure 4). This network demonstrated that several drugs and sex hormones could influence the expression of HMGB2, PDGFRA and HSD17B4. Green circles represent genes down-regulated in PCOS, while red circles indicate genes up-regulated in PCOS. For instance, several lipid-lowering drug elevated the expression of HSD17B4, which down-regulated in PCOS and down-expression of which indicate poor prognosis in breast cancer. Secondly, another lipid-lowering drug simvastatin suppressed the expression of PDGFRA, which up-regulated in PCOS and over-expression of which also suggest poor prognosis. Furthermore, pirinixic acid, an agonist of PPARa that controls the expression of genes involved in fatty acid utilization, restore the decreased expression of HSD17B4 and HMGB2 in PCOS. In addition, folic Acid, choline, and sex hormone (estrogen, testosterone and progesterone) and its derivatives (ethinyl estradiol) and analogs (coumestrol, diethylstilbestrol and pregnenolone carbonitrile) were also indicated in this network, as you can see in Figure 4. Androgen excess coupled with estrogen excess are well-known to contribute to pathogenesis of PCOS. Here, we discovered from the gene-drug interaction network that testosterone resulted in decreased expression of HMGB2, while estradiol and progesterone gave rise to up-regulation of PDGFRA, which is in accordance with the expression of HMGB2 and PDGFRA in PCOS.

## DISCUSSION

PCOS, as a complex endocrine and metabolic disorder, the underlying molecular mechanisms remains largely unknown. Numerous studies point towards adipose tissue and insulin resistance as the key elements in the development of the disorder, particularly in young patients with increased LH and hyperandrogenism (a consequence of hyperinsulinemia) [15–17], while other researchers would suggest an over-active GnRH pulse generator as the primary abnormality [18].

Our study report here is the first to overlap genes differently expressed in muscle tissue of obese insulin-resistant PCOS women and genes exert their roles in breast cancer prognosis to find the common key genes and potential therapeutic targets of PCOS and breast cancer.

Skeletal muscle accounts for the majority of glucose metabolism suggesting an important role in metabolic disorder. In this study, we compare gene expression of muscle tissue in obese insulin-resistant PCOS women and their age- and body mass index- matched healthy control women in two GSE datasets. The synchronously expressed genes in both datasets were then used to perform prognosis analysis in breast cancer patients. Fifty-three genes were found, which we hypothesized to be the potent key genes contribute to the onset of PCOS.

Excess androgens and estrogens are believed to contribute to the pathogenesis of PCOS, although the specific mechanism is still unclear [2]. According to our results, HMGB2, PDGFRA and HSD17B4 which involved in development of primary male sexual characteristics are 3 of the 53 potent key genes in pathogenesis of PCOS. HSD17B is a group of ethanol dehydrogenase, which controls the last step in formation of androgens. HSD17B4 involves in the degradation of androstenediol to testosterone [19, 20], down expression of which in PCOS may result in the accumulation of androstenediol in blood circulation, which may be partly responsible for androgen excess induced PCOS phenotype. We also discovered that PDGFRA, a cell surface tyrosine kinase receptor involved in mesenchymal cell proliferation and male-specific mesonephric cell migration [21], is found up-regulated in obese PCOS women, which may contribute to the hyperandrogenism and appearance male characters.

Dysregulation of fatty acid metabolism is a typical manifestation of PCOS syndrome, and targeting dyslipidemia appear to have a more beneficial effect [22]. We demonstrated that through gene-drug interaction network many available drugs that suggested to restore the dysregulation of lipid metabolism are efficient in correcting the abnormal gene expression of HMGB2, PDGFRA and HSD17B4 in PCOS. Thus, our results suggested the indicative value of HMGB2, PDGFRA and HSD17B4 to be the important genes in pathogenesis of PCOS on one hand, and on another hand, the hypolipidemia drug detected from our network could provide more choices for treatment of disorders of lipid metabolism in obese PCOS women.

The molecular mechanism of polycystic ovarian syndrome, which is short for PCOS, is still unclear at present, due to heterogeneity and confounding factors. Here we first combined the differentially expressed genes in PCOS and genes that have prognostic value of breast cancer together to explore common key genes in pathogenesis of PCOS and breast cancer, considering the similar pathogenesis of hyperandrogenism in both PCOS and breast cancer. The strategy of taking the differential expressed genes in PCOS and the genes of prognostic value of a certain disease that related to PCOS together may provide a new way to screen key genes associated with the onset of a PCOS in the future. In addition, these key genes would be potential therapeutic targets for both PCOS and breast cancer.

## MATERIALS AND METHODS

### Ethics statement

We are entitled to freely use the Gene Expression Omnibus (GEO) datasets in National Center for Biotechnology Information databases by meeting with its freedom-to-publish criteria. The Research Ethics Committee in Peking University Third Hospital waived the requirement for ethical approval of this study because the registry is a de-identified database.

### Selection of differently expressed genes in PCOS

Two publicly available datasets were downloaded to identify the differently expressed genes in polycystic ovarian syndrome. The cDNA microarray of GSE8157 and GSE6798 were all performed using the HG-U133 Plus 2.0 expression array from Affymetrix. Each dataset contains more than 10 samples in both PCOS patients group and healthy control group respectively.

GSE8157 contains expression data from skeletal muscle of 10 obese women with PCOS metabolically characterized by a euglycemic-hyperinsulinemic clamp, and 13 obese healthy control women. GSE6798 contains expression data of skeletal muscle from 16 obese women with PCOS and 13 age- and body mass index- matched obese healthy control women, metabolically characterized by euglycemic-hyperinsulinemic clamp and indirect calorimetry. Criteria in both datasets included irregular periods with cycle length > 35 days during last year, elevated free testosterone levels ( > 0.035 nmol/l), and/or hirsutism (total Ferriman-Gallwey score > 7), and the absence of diabetes, hypertension, hyperprolactinemia, hypothyroidism, and adrenal enzyme defects. Healthy control volunteers had regular menses, normal glucose tolerance, and no family history of diabetes. In GSE8157, all PCOS patients accepted to withdraw oral contraceptives for more than 3 months before using barrier contraception combined with spermatocidal cream during the study period. In GSE 6798, no subjects were taking medicines known to affect hormonal or metabolic parameters.

Differently expressed genes in GSE8157 were selected using the criteria of fold change more than 1.5 both up-regulated and down-regulated with *p*-value less than 0.05, and further verified by synchronously expressed in GSE 6798 with *p*-value of less than 0.05.

### Identification of hub genes in pathogenesis of PCOS by survival analysis

The differently expressed genes of PCOS using both GSE 8157 and GSE6798 were further conducted for Kaplan-Meier analysis of relapse-free survival (RFS). Our merged dataset containing 3554 breast cancer patients in different stages were included for relapse-free survival analysis. Specific methods for building this dataset was described previously [23]. Hazard ratio is to weigh the relative influence of genes on prognosis. Genes with hazard ratio of more than 1 was deemed as potential oncogene, high expression of which correlated with bad prognosis of breast cancer. While genes with hazard ratio of less than 1 was considered as potential tumor suppressor gene, high expression of which correlated with good prognosis. Genes differently expressed in both GSE 8157 and GSE6798 and of prognostic value of breast cancer progression were deemed as hub genes which may play an important role in pathogenesis of PCOS.

### Generation of genes-drug interaction network

The Comparative Toxicogenomics Database (CTD) [24] was conducted to explore the genes-drug interaction network. To be specific, genes was searched in the CTD database for drugs or chemicals that could result in their decreased or increased expression. Then sex hormone related chemicals and lipid-lowering drugs were selected based on their relationship with polycystic ovarian syndrome.

### Statistical analysis

Clinical prognosis of breast cancer and gene expression profiling were analyzed using standard statistical tests including the Logrank test and unpaired *t*-test respectively. Differently expressed genes with *p*-value of less than 0.05 was considered as of significance. Hazard ratio of either more than 1 or less than 1 with *p*-value of less than 0.05 was deemed as of prognostic value in breast cancer progression.

## SUPPLEMENTARY MATERIALS TABLE




