# Supplementary material for: PDGFRA, HSD17B4 and HMGB2 are potential therapeutic targets in polycystic ovarian syndrome and breast cancer
Source: Oncotarget. 2017 May 13;8(41):69520–6. doi: 10.18632/oncotarget.17846 (PMC5642496; doi:10.18632/oncotarget.17846)
Supplement: Supplementary file 1 [file oncotarget-08-69520-s001.pdf]

PDGFRA, HSD17B4 and HMGB2 are potential therapeutic targets in polycystic ovarian syndrome and breast cancer

Supplementary Materials

Supplementary Table 1: Potent key genes in pathogenesis of PCOS. See Supplementary_Table 1
